# Long non-coding RNA BRCAT54 sponges microRNA-21 in vestibular schwannoma to suppress cell proliferation

**DOI:** 10.1080/21655979.2022.2031410

**Published:** 2022-02-09

**Authors:** Gang Xiao, Bin Huang, Ming Guo, Chaoxin Long, Pingan Li, Bin Zhong, Chuncheng Guan

**Affiliations:** Neurosurgery Department, North Guangdong People’s Hospital, Shantou University/Medical College, Shaoguan City, PR. China

**Keywords:** Vestibular schwannoma, vestibular nerve, BRCAT54, miR-21, acoustic neuroma, cell proliferation

## Abstract

BRCAT54 (also known as MRPS30 divergent transcript) is an anti-tumor long non-coding RNA (lncRNA) in lung cancer, while its role in vestibular schwannoma (VS) is unclear. We predicted that BRCAT54 could interact with microRNA (miR)-21, which suppresses VS cell proliferation. This study was then carried out to study the interaction between BRCAT54 and miR-21 in VS. A total of 56 VS samples and 42 normal vestibular nerve (VN) samples were included in this study. The expression of BRCAT54 and miR-21 in these samples were analyzed with RT-qPCR. Subcellular location of BRCAT54 in primary VS cells was analyzed by subcellular fractionation assay. The direct interaction between BRCAT54 and miR-21 was analyzed through RNA pull-down assay. Overexpression assay was performed to explore the interaction between BRCAT54 and miR-21. The role of BRCAT54 and miR-21 in primary VS cell proliferation was analyzed using BrdU assay. We found that BRCAT54 was downregulated in VS samples than that in VN samples, while miR-21 was upregulated in VS samples. BRCAT54 and miR-21 were not closely correlated. BRCAT54 was detected in both nuclear and cytoplasm samples, and BRCAT54 directly interacted with miR-21. However, BRCAT54 and miR-21 did not affect the expression of each other. BRCAT54 suppressed primary VS cell proliferation and inhibited the role of miR-21 in promoting cell proliferation. Therefore, BRCAT54 may sponge miR-21 to suppress cell proliferation in VS.

## GRAPHICAL ABSTRACT

**Figure:**
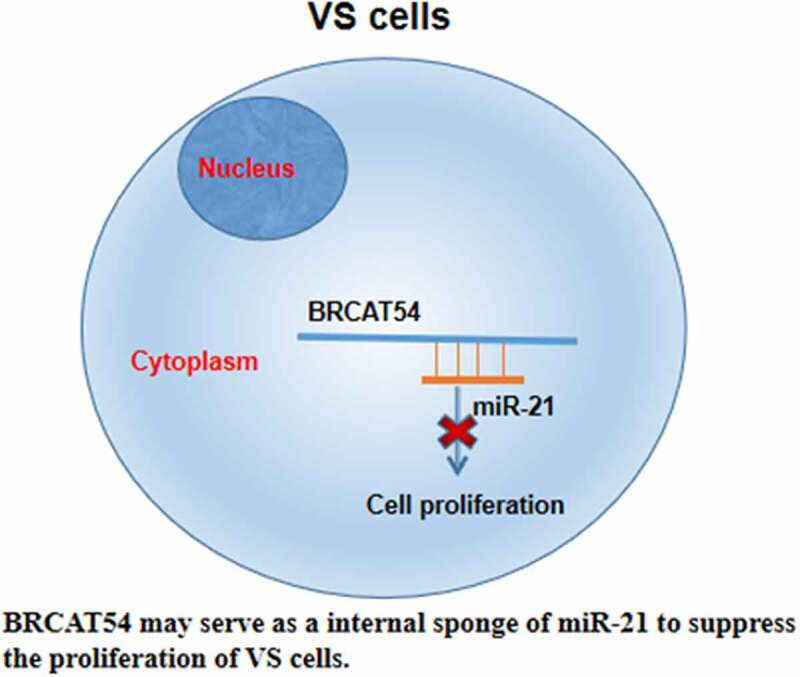

## Introduction

Vestibular schwannoma (VS), also refers to acoustic neuroma, is a type of slow-growing and benign tumor originates from vestibular nerve, which links inner ear and brain [1]. It has been well established that branches of vestibular nerve are responsible for hearing and detecting balance [2,3]. Therefore, the development of VS may lead to hearing loss and balance problems [2–4]. Although VS was considered as a rare disease and only affects about 1 out of 1 million people, a rising incidence has been reported in recent years [5,6]. In effect, in some populations the incidence of VS can be as high as 20 per million [5,6]. At present, VS is usually treated with surgical resection, radiation therapy, and observation [1,7–9]. However, muscle weakness caused by VS cannot be fully recovered after these therapies and the hearing loss is usually permanent [1,8,9].

VS in most cases is not lethal [10,11]. However, with the growth of tumors, nearby brain structures will be eventually pressed, leading to deaths [10,11]. Therefore, controlling the growth of VS is critical for the survival of VS patients. Previous studies have identified a big number of molecular factors involved in this disease [12,13]. In effect, regulating the expression of certain factors may contribute to the treatment of VS. Long non-coding RNAs (lncRNAs) have no capacity of protein-coding, but affect protein synthesis to participate in human diseases [14]. Previous studies have characterized the functions of a considerable number of lncRNAs in different types of cancer [15,16]. However, their roles in VS are largely unknown. BRCAT54 (also known as MRPS30 divergent transcript) is an anti-tumor lncRNA in lung cancer [17]. We predicted that BRCAT54 could interact with microRNA (miR)-21, which suppresses VS cell proliferation [18]. Therefore, it will be reasonable to hypothesize that BRCAT54 may interact with miR-21 to participate in VS. We then studied the interaction between BRCAT54 and miR-21 in VS.

## Materials and methods

### Human specimens

The Institutional Review Board of North Guangdong People’s Hospital, Shantou University/Medical College has approved this study. Human specimens used in this study included unilateral sporadic VS samples from 56 VS patients (26 females and 30 males, 48.3 ± 7.8 years old). VS samples were collected during surgical resections, which were performed either through translabyrinthine or retrosigmoid approach. For the control group, normal vestibular nerve (VN) samples were collected form 42 controls (19 females and 23 males, 48.5 ± 7.5 years old) during vestibular neurectomy. All tissue samples were collected through a sterile way and stored in a liquid nitrogen before use. All participants signed the informed consent.

### Primary VS cell preparation and cultures

Resected VS tumors were used to prepare small pieces, which were subjected to collagenase (2 mg/ml, Sigma) and trypsin (0.25%, Sigma) digestion at 37°C for 1 h. After adding FBS to 10%, samples were centrifuged at 800 rpm for 3 min. Cell pellets were resuspended and transferred to a 4-well culture plate. DMEM medium containing 1 mg/ml bovine insulin (Sigma) and 10% FBS was used to cultivate cells until confluence reached 50–70%. Cells were then maintained in serum-free medium prior to use. More than 90% of these cells were S100-positive. Cell culture was performed in an incubator with temperature, humidity, and CO_2_ set to 37°C, 95%, and 5%, respectively.

### Cell transfections

Overexpression of BRCAT54 and miR-21 was achieved in cells by transfecting cells with BRCAT54 vector (pcDNA3.1) and mimic of miR-21. Transfections were performed using the FuGENE transfection reagent (Life Technologies) following the manufacturer’s instructions. Briefly, 10^7^ cells were incubated with 100 nM miRNA or 10 nM vector for 6 h, followed by washing for three times with fresh medium to reduce cytotoxicity. Cell transfections were confirmed prior to use.

### RNA preparation

The Monarch Total RNA Miniprep Kit (NEB) was used to prepare total RNAs. Samples were first mixed with DNA/RNA Protection Reagent, Prot K Reaction Buffer and Prot K, followed by incubation at 55°C for 5 min. Following centrifugation at 12,000 g for 2 min, supernatant was transferred to RNase-free tubes. After that, cell lysis buffer was added. gDNA removal were performed using gDNA removal column through centrifugation. RNA purification column was then applied to perform RNA binding. After washing with washing buffers, RNA samples were eluted with RNase-free water.

### RNA analysis and RT-qPCR

RNA integrity was analyzed by urea-PAGE gel electrophoresis and 2100 Bioanalyzer. All RNA samples showed a RIN value higher than 2.0. The quality and concentration of RNA samples were detected by Bioanalyzer. RNA samples were added with RNase-free water to adjust the concentration to about 2,000 ng/µl. The SSRT III system (Invitrogen) was then used to prepare cDNA samples through reverse transcriptions with 1 µl RNA sample as template. With 1 µl cDNA sample as template, qPCR was performed to assay the expression of BRCAT54 and miR-21 using 18S rRNA as an internal control. Relative gene expression levels were determined by analyzing Ct values with the 2^− ΔΔCT^ method [19].

### Nuclear-cytoplasmic fractionation assay

Preparation of nuclear and cytoplasm samples from cells was performed using Cell Fractionation Kit (ab109719, Abcam). About 10^7^ cells were harvested and resuspended in cell lysis buffer. Incubation with lysis buffer was performed on ice for about 30 min, followed by performing centrifugation in cold room for 20 min at 1200 g to separate cytoplasm fraction, which was the supernatant. Cell pellet was further incubated with nuclear lysis buffer on ice for 20 min. After that, two fractions were subjected to RNA extraction and purification. After that, cDNA samples were prepared and PCR was performed to amplify BRCAT54 with GAPDH as a cytoplasmic control.

### RNA-RNA pulldown assay

The in vivo interaction between BRCAT54 and miR-21 was analyzed using biotin-labeled RNA transcripts of both negative control (NC) RNA and BRCAT54, which were prepared through in vitro transcriptions with T7 reverse transcriptase (NEB). RNA transcripts were first purified using Monarch® RNA Cleanup Kit (NEB), following by labeling the 3ʹend with Pierce™ Biotin 3’ End DNA Labeling Kit. Monarch® RNA Cleanup Kit (NEB) was used to purified the labeled RNAs. The labeled RNAs, including bio-NC and bio-BRCAT54, were transfected into cells. Cells were gathered and cell lysis was performed. Magnetic beads were then used to incubate with cell lysates to pull-down RNA complex. Finally, RNA samples were prepared, followed by RT-qPCR to detect the expression of miR-21 in pull-down samples.

### Dual luciferase reporter assay

Dual-luciferase reporter assay was performed as previously described [20]. Luciferase vector of BRCAT54 was constructed using psiCHECK-2 vector (Promega) as the backbone. Luciferase vector of MIR17HG was co-transfected with NC miRNA (NC group) or miR-21 mimic (miR-21 group). Luciferase activity was measured 48 h later.

### BrdU assay

The incorporation of BrdU into cells was determined to reflect cell proliferation [21]. Briefly, cells were harvested after transfections and transferred to a 96-well plate with 6,000 cells in 0.1 ml medium per well. Three replicate wells were set for each experiment. BrdU solution was added into each well to reach 10 mM, followed by cell culture for another 4 h. After that, cells were stained with anti-BrdU antibody for 2 h, followed by incubation with secondary antibody for 2 h. After that, tetramethyl benzidine substrate was added, followed by incubation for 1 h. Then cell proliferation was analyzed by measuring OD values at 450 nm.

### Statistical analysis

Image preparation and data analyses were performed using GraphPad Prism version 6 software. Differences between groups were explored using two-tailed Student’s T-tests. Correlations were explored with Pearson’s correlation coefficient. P < 0.05 was considered significant difference.

## Results

### Expression analysis of BRCAT54 and miR-21 in VS and normal VN samples

**Figure 1.**
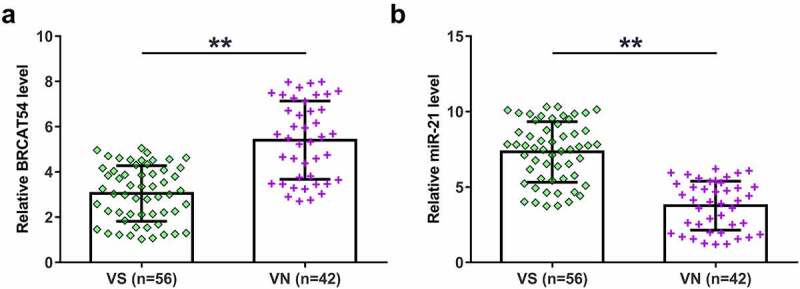
Expression analysis of BRCAT54 and miR-21 in VS and normal VN samples. VS (n = 56) and normal VN (n = 42) samples collected in this study were subjected to the extraction of total RNAs, which were used to prepare cDNA samples. qPCR were performed with cDNA samples as template to determine the expression of BRCAT54 (a) and miR-21 (b). Data presented were average values of three technical replicates. **, *p* < 0.01.

Functional characterization requires the analysis of differential gene expression. Therefore, VS (n = 56) and normal VN (n = 42) samples collected in this study were subjected to the extraction of total RNAs samples and qPCRs to detect the expression of BRCAT54 and miR-21. The expression levels of BRCAT54 were decreased in VS samples than that in VN samples (Figure 1(a), *p* < 0.01), while the expression levels of miR-21 were increased in VS samples (Figure 1(b), *p* < 0.01). These data suggested that BRCAT54 and miR-21 were possibly involved in VS.

### Correlations between BRCAT54 and miR-21

**Figure 2.**
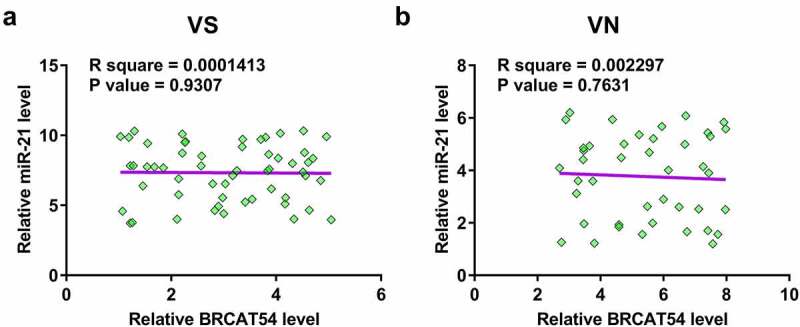
Correlations between BRCAT54 and miR-21. Correlations between BRCAT54 and miR-21 across both VS (a) and VN (b) samples were analyzed using Pearson’s correlation coefficient. Data presented were average values of three technical replicates.

Pearson’s correlation coefficient was used to explore the potential correlation between BRCAT54 and miR-21 across both VS and VN samples, which may indicate possible crosstalk between them. The results showed that BRCAT54 and miR-21 were not closely correlated across VS samples (Figure 2(a)). In addition, no significant correlation was observed between them across VN samples as well (Figure 2(b)). Therefore, BRCAT54 was unlikely a target of miR-21.

### Analysis of the location of BRCAT54 in VS cells and its direct interaction with miR-21

**Figure 3.**
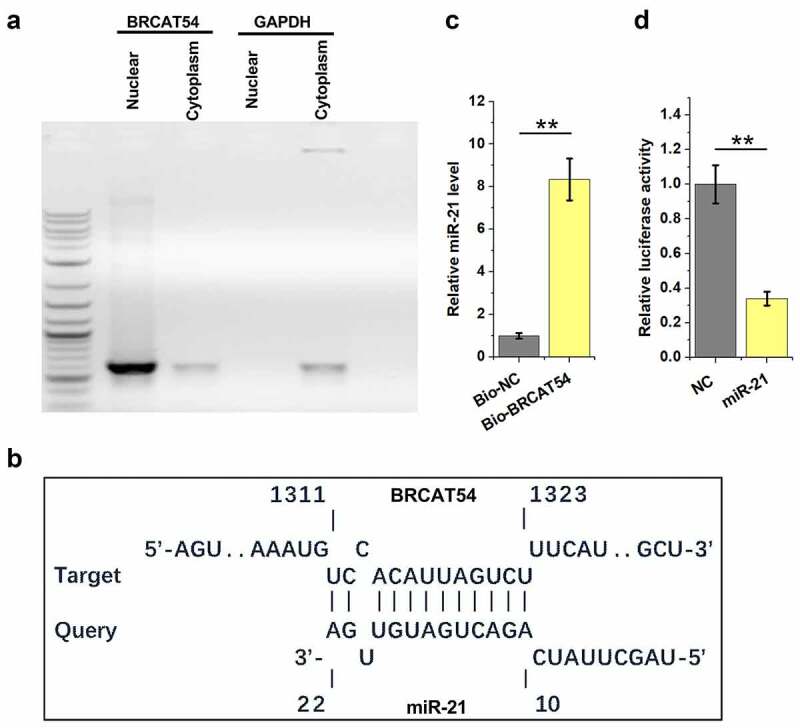
Analysis of the location of BRCAT54 in VS cells and its direct interaction with miR-21. Nuclear and cytoplasm samples of VS cells were prepared, followed by RT-PCR to detect BRCAT54 (a). IntaRNA 2.0 was applied to predict the direct interaction between BRCAT54 and miR-21 (b). RNA-RNA pulldown assay was applied to confirm their direct interaction (c). Dual luciferase reporter assay was performed to further verify the direct interaction between them (d). **, *p* < 0.01.

Subcellular location of RNAs determines their function. Therefore, nuclear and cytoplasm samples of VS cells were prepared, followed by RT-PCR to detect BRCAT54. The results showed that BRCAT54 could be detected in both nuclear and cytoplasm samples (Figure 3(a)). IntaRNA 2.0 was applied to predict the direct interaction between BRCAT54 and miR-21. The prediction showed that BRCAT54 and miR-21 could form base paring (Figure 3(b)). RNA–RNA pulldown assay was performed to confirm their direct interaction. The results showed that the expression levels of miR-21 were significantly higher in Bio-BRCAT54 pulldown samples compared to that in Bio-NC pull-down samples, which confirmed the direct interaction between them (Figure 3(c), *p* < 0.01). Dual-luciferase reporter assay was performed to further confirm the direct interaction between them. Compared to NC group, much lower luciferase activity was observed in miR-21 group (Figure 3(d), *p*< 0.01). Therefore, BRCAT54 might be able to bind to miR-21.

### The role of BRCAT54 and miR-21 in regulating the expression of each other

**Figure 4.**
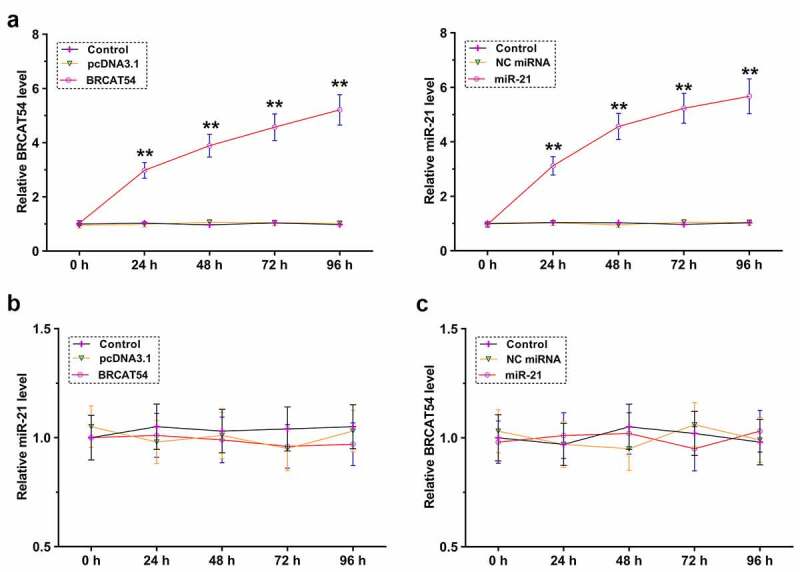
The role of BRCAT54 and miR-21 in regulating the expression of each other. Primary VS cells were overexpressed with BRCAT54 or miR-21, and the overexpression was confirmed every 24 h until 96 h (a). RT-qPCR were performed to detect the expression of miR-21 in BRCAT54-overexpressing cells (b) and the expression of BRCAT54 in miR-21-overexpressing cells (c). **, *p* < 0.01.

The direct interaction between them may indicate the potential crosstalk between them. To explore this, primary VS cells were overexpressed with BRCAT54 or miR-21, and the overexpression was confirmed every 24 h until 96 h (Figure 4(a), *p*< 0.01). RT-qPCR was performed to detect the expression of miR-21 in BRCAT54-overexpressing cells (Figure 4(b)) and the expression of BRCAT54 in miR-21-overexpressing cells (Figure 4(c)). BRCAT54 and miR-21 did not affect the expression of each other. Therefore, BRCAT54 was unlikely a target of miR-21.

### Analysis of the role of BRCAT54 and miR-21 in the proliferation of VS cells

**Figure 5.**
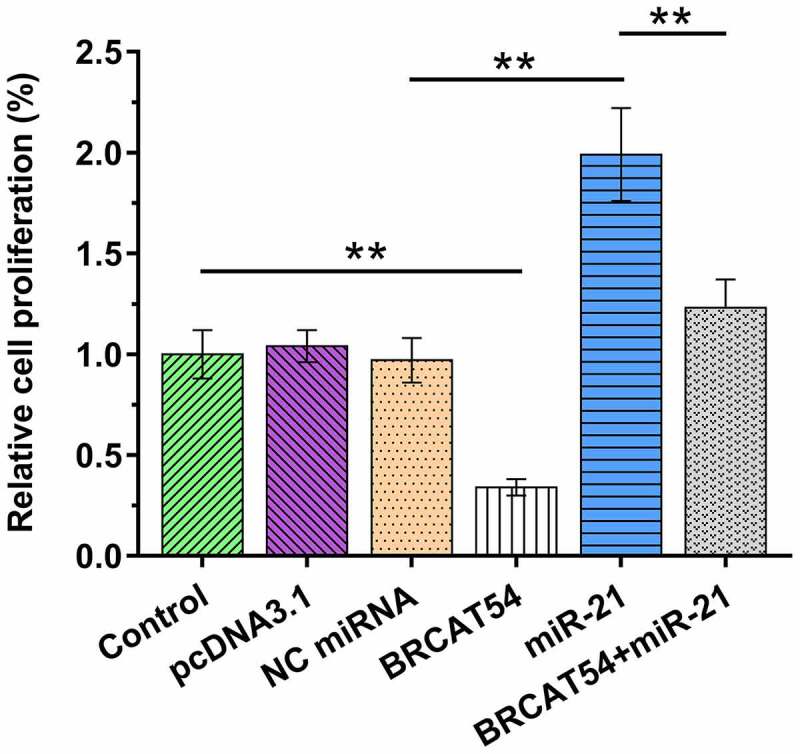
Analysis of the role of BRCAT54 in miR-21 in the proliferation of VS cells. Cells overexpressed with BRCAT54 and/or miR-21 were subjected to cell proliferation analysis through BrdU assays. Data presented were mean±SD values of three biological replicates. **, *p* < 0.01.

Cell proliferation determines tumor growth. Cells overexpressed with BRCAT54 and/or miR-21 were therefore subjected to BrdU assays to analyze the role of BRCAT54 and miR-21 in the proliferation of VS cells. BRCAT54 suppressed primary VS cell proliferation. In contrast, miR-21 increased cell proliferation. Moreover, BRCAT54 inhibited the role of miR-21 in promoting cell proliferation (Figure 5, *p*< 0.01). Therefore, BRCAT54 may suppress cell proliferation in VS by sponging miR-21.

## Discussion

In this study, we investigated the participation of BRCAT54 in miR-21 in VS. We observed altered expression of BRCAT54 and miR-21 in VS. Functional assays were then performed and the results showed that BRCAT54 may serve as an internal sponge of miR-21 to suppress the proliferation of VS cells.

In a recent study, Yang *et al*. identified a novel lncRNA named BRCAT54 with tumor suppressive effects on non-small cell lung cancer (NSCLC) [17]. They showed that BRCAT54 was downregulated in both NSCLC tumors and cells, and BRCAT54 could positively regulate the growth of NSCLC tumors by directly binding to RPS9, thereby by regulating the JAK-STAT pathway [17]. The role of BRCAT54 in other biological or physiological processes is unclear. In this study, we observed the downregulation of BRCAT54 in VS compared to that in normal VN samples. Moreover, overexpression of BRCAT54 significantly suppressed the proliferation of primary VS cells. Therefore, BRCAT54 may participate in VS by suppressing cell proliferation, and overexpression of BRCAT54 may serve as a potential target to treat VS by suppressing tumor growth. However, this study only performed *in vitro* cell model experiments. The function of BRCAT54 in VS should be verified by *in vivo* animal model experiments.

MiR-21 is upregulated in VS and promotes VS cell survival and proliferation [18]. The present study confirmed the upregulation of miR-21 in VS and the promoting effects on VS cell proliferation. However, the upstream regulators of miR-21 are unclear. In this study, we also observed that BRCAT54 could directly interact with miR-21, while they did regulate the expression of each other in primary VS cells. In addition, BRCAT54 and miR-21 were not closely correlated across VS and VN samples. Therefore, BRCAT54 was unlikely a target of miR-21. It is known that lncRNAs may serve as the internal sponge of miRNAs to suppress their function, but may not affect their expression [22]. Since BRCAT54 was detected in cytoplasm, we speculated that BRCAT54 could sponge miR-21 in cytoplasm to suppress its roles in promoting VS cell proliferation.

## Conclusion

In conclusion, BRCAT54 is downregulated in VS and miR-21 is upregulated in VS. In addition, BRCAT54 may serve as an internal sponge of miR-21 to suppress the proliferation of VS cells.

## Data Availability

The analyzed data sets generated during the study are available from the corresponding author on reasonable request.
